# Incidence and Outcomes of Pericardial Effusion After Congenital Heart Surgery

**DOI:** 10.1177/21501351251322876

**Published:** 2025-09-09

**Authors:** Mario O’Connor, Carlos M. Mery, Neil M. Venardos, Jeremy Affolter, Charles Fraser, Andrew Well

**Affiliations:** 1 Texas Center for Pediatric and Congenital Heart Disease, 377659 The University of Texas at Austin Dell Medical School, Austin, TX, USA; 2 Congenital Heart Disease, 5718 Vanderbilt University, Nashville, TN, USA; 3 Congenital Cardiac Surgery, 173868 Oklahoma Children's Hospital, Oklahoma City, OK, USA

**Keywords:** pericardium (includes everything related), pericardial effusion, congenital heart disease (CHD), congenital heart surgery, complications

## Abstract

**Introduction:** Pericardial effusion (PCE) represents a significant postoperative complication following congenital heart surgery (CHS), contributing to more complex postoperative care and heightened morbidity. In this study, we aim to elucidate the risk factors contributing to PCE development post-CHS through analysis of data from a nationwide, multi-institutional database. **Methods:** Review of the Pediatric Health Information System Database from January 1, 2004, to December 30, 2023. All patients under the age of 18, who underwent a Society of Thoracic Surgeons benchmark procedure, were included. **Results:** A total of 66,695 surgeries were identified with 29,363 (44%) female, 35,084 (53%) non-Hispanic white, and median age at surgical admission of 5.1 [interquartile range: 2.0-14.1] months. Pericardial effusion occurred in 2,672 (4%) patients during the postoperative hospitalization, with the Norwood procedure having the highest incidence (245/2,726, 8.9%). Multivariable analysis revealed the Norwood procedure as having the highest adjusted odds of PCE (odds ratio [OR]: 2.34, 95%CI: 1.98-2.75, *P* < .001) when compared with isolated ventricular septal defect repair. Pericardial effusion was associated with a 16% (95%CI: 9.8-12.3, *P* < .001) increase in length of stay and increased mortality (OR: 1.84, 95%CI: 1.50-2.25, *P* < .001). Furthermore, 647 (1%) patients, out of 65,442 who survived to index hospital discharge, were readmitted due to PCE within 90 days of discharge. Fontan patients had the highest incidence of readmission (138/7,873, 1.7%) and increased adjusted odds for readmission (OR: 1.77, 95%CI: 1.37-2.28, *P* < .001). **Conclusions:** Incidence of PCE after CHS is low at 4%. However, certain procedures have an incidence as high as 8.9%. Pericardial effusion poses significant postoperative clinical challenges with increased mortality and resource utilization. Vigilant monitoring and targeted interventions in high-risk patients are essential for mitigating the impact of PCE on postoperative outcomes.

## Introduction

Pericardial effusion (PCE) represents a significant postoperative complication following congenital heart surgery (CHS).^1–3^ It contributes to complex postoperative care, heightened morbidity, and an increased rehospitalization rate.^4^ The exact pathophysiology of PCE remains unclear; however, it is known to be part of the post-pericardiotomy syndrome.^5,6^ This syndrome is an immune-mediated inflammatory reaction that occurs as a result of trauma to the pericardium.^7^

The reported incidence of PCE following CHS varies widely, ranging from 1% to 69%.^1,2,8^ This substantial range reflects differences in patient populations, procedural types, definitions of PCE, and diagnostic practices across reports. Studies focusing on high-complexity procedures or high-risk patient groups often report higher rates, while those including broader, lower-risk cohorts report lower rates. Furthermore, variations in how PCE is diagnosed may also contribute to this disparity.

Numerous risk factors for PCE have been identified, including longer cardiopulmonary bypass time, younger age, genetic syndromes, male gender, repair of atrial septal defects (ASDs), and whether the procedure is a first-time cardiac surgery. However, these findings are often derived from studies encompassing a wide spectrum of procedures and patient complexities, making it difficult to draw procedure-specific conclusions or benchmark institutional performance.^1,8^

Given the variability in procedural types and complexities, there remains a critical need for focused analyses targeting the Society of Thoracic Surgeons (STS) benchmark procedures. These procedures—including ventricular septal defect (VSD) repair, arterial switch operation (ASO), ASO + VSD repair, tetralogy of Fallot (TOF) repair, Norwood procedure, Glenn procedure, Fontan procedure, off-pump coarctation repair, truncus arteriosus repair, and atrioventricular septal defect (AVSD) repair—are standardized interventions used to assess surgical quality and performance in CHS.^9^

By focusing on STS-benchmark procedures, this article aims to systematically assess the incidence and outcomes of PCE within a standardized framework. This approach facilitates comparisons across different procedures and helps to identify variations in patient outcomes.

## Patients and Methods

### Data Source

This study is a retrospective analysis utilizing data from the Pediatric Health Information System (PHIS) database, spanning January 1, 2004, to December 30, 2023. Managed by the Children's Hospital Association (CHA), PHIS contains administrative and billing information from 49 pediatric hospitals, representing approximately 20% of pediatric hospitalizations across the United States. The data submitted to PHIS is deidentified and subjected to thorough quality assurance processes. Each patient is assigned a unique identifier for every hospital visit, allowing for longitudinal tracking within the same hospital, although not across other CHA facilities. Pediatric Health Information System data include primary diagnoses, along with up to 41 additional diagnoses, as well as primary procedures and up to 41 additional procedures.

### Study Population

All patients in the PHIS database with an *International Classification of Disease* (ICD) code consistent with congenital heart disease and underwent an STS-benchmark congenital cardiac surgery procedure were included in the main cohort. *International Classification of Disease* codes used for disease and procedure identification have been previously validated.^10^ The following were excluded from analysis: events with missing information on the type of admission, sex, age, race, ethnicity, length of stay (LOS), discharge status, admitting diagnosis, and/or principal diagnosis.

Pericardial effusion was defined in our cohort as those with an ICD diagnosis code for PCE, hemopericardium, cardiac tamponade, and/or those with a procedure code for pericardiocentesis or percutaneous drainage of the pericardium/mediastinum during the hospitalization for the surgical procedure but after benchmark operation (Supplemental Table 1). Patients were followed for 90 days after discharge from index hospitalization, and the same definition was used to define readmission involving a PCE. Further diagnoses, procedures, and outcomes were identified using ICD codes (Supplemental Table 1).

### Study Outcomes

The primary aim of the study was to assess the incidence and outcomes of PCE after STS benchmark congenital cardiac surgery procedures. Demographics collected included age, sex, race, ethnicity, and insurance type. Insurance was grouped into Government (Medicare, Medicaid, Tricare, etc), Private, and Other (Charity, other payor, unknown). The study period was divided into three eras (2004-2009, 2010-2015, and 2016-2023). Hospitalization outcomes included LOS, need for intervention, and in-hospital mortality, all provided by PHIS. Additionally, readmissions within 90-day postdischarge were analyzed.

### Statistical Analysis

Descriptive statistics were reported for demographics, clinical characteristics, and outcomes. Categorical variables are reported as n (%). Length of stay is reported in median [interquartile range (IQR)] days. Chi-square and Fisher exact test were utilized to analyze noncontinuous variables. Kruskal-Wallis test was utilized to analyze LOS comparison between groups. Multivariable analyses including linear and logistic multivariable regression were utilized to assess associations with PCE and outcomes. Statistical analyses were performed using R and RStudio.^11^

## Results

### Study Population and Demographics

From January 1, 2004, through December 30, 2023, there were 66 695 STS-benchmark procedures. Of this cohort, 29,363 (44%) were female, 35,084 (53%) were white non-Hispanic, 26,026 (39%) had private insurance, and a median age at index admission of 5.1 [IQR: 2.0-14.1] months. A total of 2,672/66,695 (4%) patients were identified as having PCE during index operation admission. Differences were observed in age and gender with patients developing PCE being younger (4.1 [IQR: 0.1-8.9] months vs 5.2 [IQR: 2.1-14.3] months, *P* < .001 and having a higher proportion of female patients (n = 1,236/2,672 (46%) vs n = 28,127/64,023 (44%), *P* = .018). The PCE group had a higher proportion of low-birth weight patients (n = 167/2,672 (6%) vs n = 2,454/64,023 (4%), *P* < .001), Trisomy 21 patients (n = 307/2,672 (11%) vs n = 6,469/64,023 (10%), *P* = .022), and DiGeorge patients (n = 84/2,672 (3%) vs n = 1,499/64,023 (2%), *P* = .009) (Table 1).

**Table 1.** Demographics.

Variable	Overall (n = 66 695)	PCE (n = 2,672) (4%)	Non-PCE (n = 64 023) (96%)	*P* value
Age				
Age at surgery (months), median [IQR]	5.1[2.0-14.1]	4.1[0.1-8.9]	5.2[2.1-14.3]	<.001
Female	29 363(44)	1,236(46)	28 127(44)	.018
Race				
White Non-Hispanic	35 084(53)	1,339(50)	33 745(53)	.058
Hispanic	11 712(18)	507(19)	11 205(18)	
Black	8,538(13)	364(14)	8,174(13)	
Other	8,524(13)	356(13)	8,168(13)	
Missing	2,837(4)	106(4)	2,731(4)	
Insurance				
Private	26 026(39)	1,025(38)	25 001(39)	<.001
Government	35 713(54)	1,508(57)	34 205(53)	
Other	4,956(7)	139(6)	4,817(8)	
Low birth weight	2,621(4)	167(6)	2,454(4)	<.001
Prematurity	3,309(5)	203(8)	3,106(5)	<.001
Trisomy 21	6,776 (10)	307(11)	6,469(10)	.022
Turner syndrome	292(0)	9(0)	283(0)	.510
Trisomy 18	89(0)	6(0)	83(0)	.173
DiGeorge syndrome	1,583(2)	84(3)	1,499(2)	.009
Era				
Era 1 (2004-2009)	22 285(33)	729(27)	21 556(34)	<.001
Era 2 (2010-2015)	23 825(36)	969(36)	22 856(36)	
Era 3 (2016-2023)	20 585(31)	974(36)	19 611(31)	
Center volume				
Low tertile	10 883(16)	503(19)	10 380(16)	.001
Middle tertile	21 375(32)	848(32)	20 527(32)	
Top tertile	34 437(52)	1,321(49)	33 116((52)	

Abbreviations: IQR, interquartile range; PCE, pericardial effusion.

Center volume was stratified by tertiles, based on the total number of CHS cases performed per center during the study period. The top tertile centers performed a median of 274.6 [IQR: 242.4-357.8] surgical cases per year. Middle tertile centers carried out a median of 176.8 [IQR: 169.7-194.8] cases per year, while bottom tertile centers had a median of 89.3 [IQR: 55.9-118.3] cases per year.

### Pericardial Effusion

**Figure 1.**
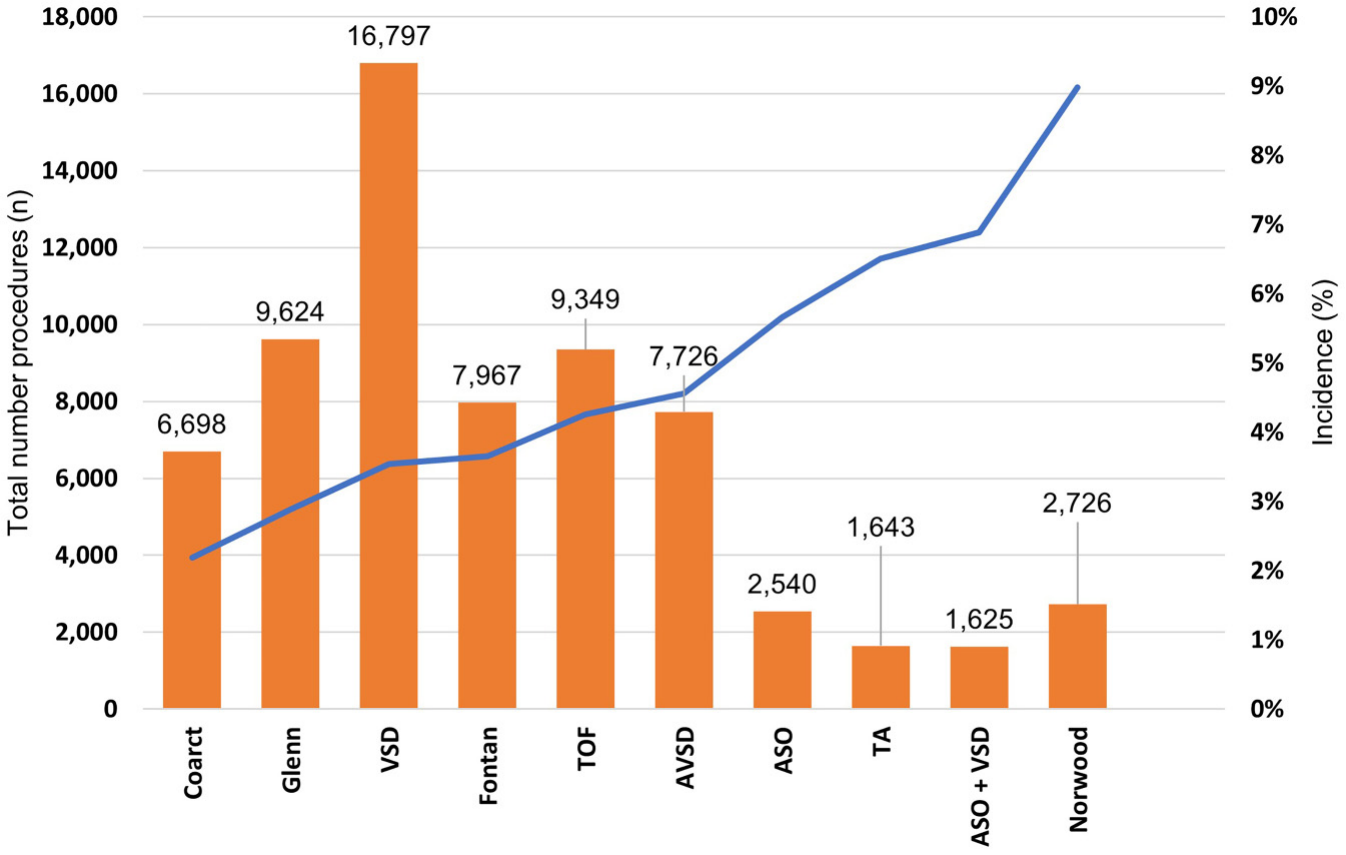
Total number of procedures and incidence of pericardial effusion by procedure. Abbreviations: ASO, arterial switch operation; AVSD, atrioventricular septal defect; Coarct, coarctation; TA, truncus arteriosus; TOF, tetralogy of Fallot; VSD, ventricular septal defect;

The three most frequent benchmark procedures were VSD repair (n = 16,797/66,695, 25%), Glenn procedure (n = 9,624/66,695, 14%), and TOF repair (n = 9,349/66,696, 14%) (Figure 1).

The procedure with the highest incidence of PCE was the Norwood procedure (n = 245/2,726, 8.9%, *P* < .001), followed by ASO + VSD (n = 112/1,625, 6.8%, *P* < .001) and truncus arteriosus repair (n = 107/1,643, 6.5%, *P* < .001). The procedure with the lowest incidence of PCE was off-pump coarctation repair (n = 147/6,698, 2.1%, *P* < .001) (Figure 1).

**Figure 2.**
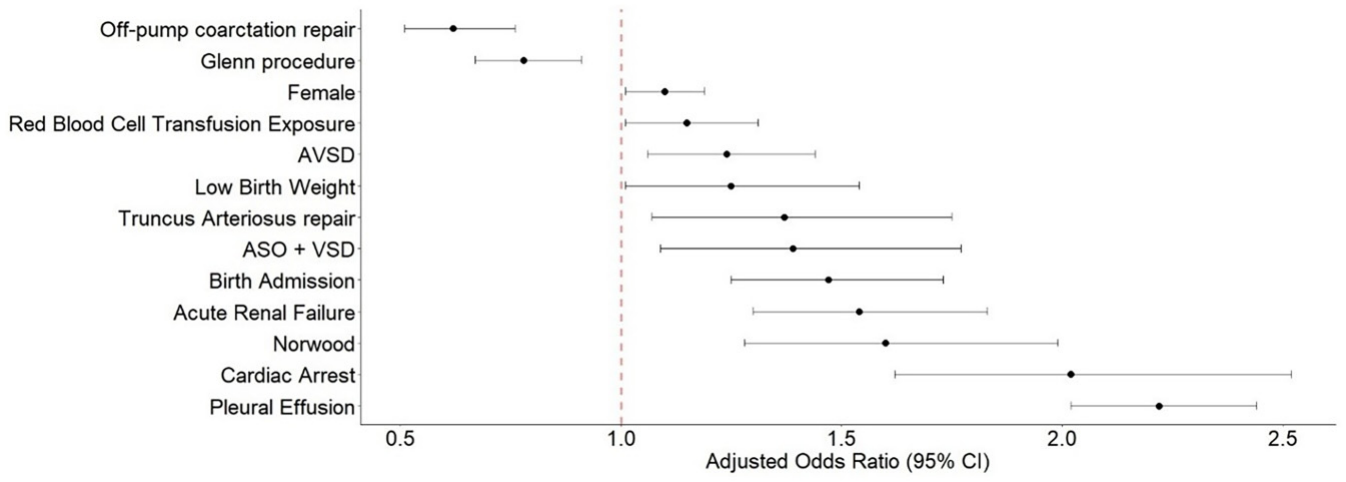
Forest plot: multivariable logistic regression model development of pericardial effusion. Reference procedure: ventricular septal defect (VSD) repair. Abbreviations: ASO, arterial switch operation; AVSD, atrioventricular septal defect.

On multivariable analysis, the Norwood procedure had the highest risk for PCE of benchmark procedures when compared with VSD repair (odds ratio [OR]: 2.34; 95% CI: 1.98-2.75, *P* < .001). ASO + VSD also had an increased risk for PCE when compared with VSD repair (OR: 1.82; 95% CI: 1.46-2.26, *P* < .001). The Glenn procedure (OR: 0.80; 95% CI: 0.69-0.93, *P* = .004) and off-pump coarctation repair (OR: 0.67; 95% CI: 0.56-0.82, *P* < .001) were associated with a decrease risk of PCE when compared with VSD repair (Figure 2).

Other independent risk factor associated with increased risk of PCE included female gender (OR: 1.10; 95% CI: 1.02-1.19, *P* = .014), low-birth weight (OR: 1.34; 95% CI: 1.09-1.65, *P* = .005), having surgery in a lower volume center (OR: 1.23; 95% CI: 1.11-1.37, *P* < .001) when compared with a high volume center. Exposure to red blood cell transfusion after surgical intervention was associated with increased risk for PCE (OR: 1.15; 95%CI: 1.01-1.31, *P* = .028). Having a diagnosis of pleural effusion (OR: 2.24; 95%CI: 2.04-2.46, *P* < .001) and a diagnosis of acute renal failure (OR: 1.61; 95% CI: 1.36-1.91, *P* < .001) were associated with increased risk for PCE (Figure 2).

### Pericardial Effusion Needing Intervention

Of the entire cohort, 526 (1%) patients underwent intervention for PCE during the index operation admission. Birth admission, low birthweight, prematurity, and DiGeorge syndrome patients were more likely to undergo intervention (Table 2).

**Table 2.** Pericardial Effusion Intervention During Index Operation Admission.

Variable	Overall (n = 66 695)	Intervention (n = 526)(1)	No intervention (n = 66 169)(99)	*P* value
Age at surgery (months), median [IQR]	5.1[2.0-14.1]	2.9[0.0-6.3]	5.1[2.1-14.2]	1
Female	29 363(44)	230(44)	29 133(44)	.924
Race				
White Non-Hispanic	35 084(53)	267(51)	34 817(53)	.809
Hispanic	11 712(18)	100(19)	11 612(18)	
Black	8,538(13)	67(13)	8,471(13)	
Other	8,524(13)	66(13)	8,458(13)	
Missing	2,837(4)	26(5)	2,811(4)	
Insurance				
Private	26 026(39)	198(38)	25 828(39)	.007
Government	35 713(54)	306(58)	35 407(54)	
Other	4,956(7)	22(4)	4,934(7)	
Birth admission	9,942(15)	178(34)	9,764(15)	<.001
Low birth weight	2,621(4)	47(9)	2,574(4)	<.001
Prematurity	3,309(5)	51(10)	3,258(5)	<.001
Trisomy 21	6,776(10)	53(10)	6,723(10)	1
Turner syndrome	292(0)	1(0)	291(0)	.733
Trisomy 18	89(0)	1(0)	88(0)	.506
DiGeorge syndrome	1,583(2)	20(4)	1,563(2)	.043
Era				
Era 1 (2004-2009)	22 285(33)	121(23)	22 164(33)	<.001
Era 2 (2010-2015)	23 825(36)	144(27)	23 681(36)	
Era 3 (2016-2023)	20 585(31)	261(50)	20 324(31)	
Center volume				
Low tertile	10 883(16)	107(20)	10 776(16)	.002
Middle tertile	21 375(32)	136(26)	21 239(32)	
Top tertile	34 437(52)	283(54)	34 154(52)	

Abbreviation: IQR, interquartile range.

On multivariable analysis, when compared with VSD repair, the Norwood procedure (OR: 2.91; 95% CI: 2.09-4.07, *P* < .001), ASO + VSD repair (OR: 2.35; 95% CI: 1.54-3.57), and ASO (OR: 1.60; 95% CI: 1.06-2.41, *P* = .023) were associated with increased odds for need of intervention. Off-pump coarctation repair had reduced odds of intervention when compared to VSD (OR: 0.49; 95% CI: 0.29-0.85, *P* = .011) (Table 3).

**Table 3.** Multivariable Logistic Regression Model Intervention Pericardial Effusion During Operation Admission.

Variable	OR	CI 95%	*P* value
Age in months	0.99	0.99-1.00	.127
Female	1.01	0.84-1.20	.889
Race			
White Non-Hispanic	Ref		
Hispanic	1.00	0.79-1.28	.940
Black	0.92	0.69-1.21	.571
Other	1.03	0.79-1.35	.804
Missing	1.03	0.68-1.56	.862
Insurance			
Private	Ref		
Government	1.13	0.93-1.37	.189
Other	0.70	0.45-1.11	.133
Low birth weight	2.07	1.38-3.08	<.001
Prematurity	1.13	0.77-1.67	.514
Trisomy 21	1.45	1.00-2.11	.049
Turner syndrome	0.79	0.10-5.77	.817
Trisomy 18	1.90	0.26-13.88	.525
DiGeorge syndrome	1.54	0.94-2.51	.080
Era			
Era 1 (2004-2009)	Ref	Ref	Ref
Era 2 (2010-2015)	1.01	0.79-1.29	.909
Era 3 (2016-2023)	1.94	1.50-2.50	<.001
Center volume			
Low tertile	1.26	1.00-1.58	.043
Middle tertile	0.75	0.61-0.93	.008
Top tertile	Ref		
Red blood cell transfusion	1.33	1.04-1.71	.022
ARF	1.24	0.88-1.74	.211
Pleural effusion	2.46	2.01-3.01	<.001
Procedures			
Norwood	2.91	2.09-4.07	<.001
ASO + VSD	2.35	1.54-3.57	<.001
Truncus arteriosus repair	1.49	0.87-2.52	.137
ASO	1.60	1.06-2.41	.023
AVSD	1.15	0.79-1.67	.447
Fontan procedure	1.20	0.83-1.72	.326
Tetralogy of Fallot repair	1.06	0.78-1.45	.668
Glenn procedure	1.30	0.95-1.77	.094
VSD repair	Ref		
Off-pump coarctation repair	0.49	0.29-0.85	.011

Abbreviations: ARF, acute renal failure; ASO, arterial switch operation; AVSD, atrioventricular septal defect; OR, odds ratio; VSD, ventricular septal defect.

Other independent risk factors associated with increased risk of intervention included low birthweight (OR: 2.07; 95% CI: 1.38-3.08, *P* < .001), Trisomy 21 (OR: 1.45; 95% CI: 1.00-2.11, *P* = .049), exposure to blood transfusion after surgical intervention (OR: 1.33; 95% CI: 1.04-1.71, *P* = .022), and having a diagnosis of pleural effusion (OR: 2.46; 95% CI: 2.01-3.01, *P* < .001) (Table 3).

### Length of Stay

Overall, total median LOS was 8 [IQR: 5-17] days, with the PCE group having longer LOS when compared to the no-PCE group (15 [IQR: 7-35] days vs 8 [IQR: 5-16] days, *P* < .001) (Supplemental Table 2). Median postoperative LOS was 7 [IQR: 5-13] days, with the PCE group having longer postoperative LOS compared to the no-PCE group (12 [IQR: 7-28] days vs 7 [IQR: 5-13] days, *P* < .001). After adjustment, PCE was associated with a 16.1% (95% CI: 14.6%-17.6%, *P* < .001) increase in total LOS (Table 4).

**Table 4.** Multivariable Linear Regression Model for Length of Stay and Multivariable Regression Model for Mortality.

	Length of stay			Mortality		
Variable	Percent difference (%)	95%CI	*P* value	OR	95%CI	*P* value
Pericardial effusion	16.08	14.57-17.61	<.001	1.84	1.50-2.25	<.001
Age in months	−0.24	−0.25 to −0.23	<.001	0.99	0.98-0.99	<.001
Female	0.93	0.40-1.45	<.001	1.21	1.08-1.37	.001
Race						
White Non-Hispanic	Ref					
Hispanic	3.14	2.39-3.89	<.001	1.02	0.86-1.21	.760
Black	2.82	1.98-4.68	<.001	1.42	1.19-1.70	<.001
Other	3.06	2.24-3.89	<.001	1.38	1.15-1.65	.004
Missing	5.01	3.66-6.38	<.001	2.17	1.72-2.74	<.001
Insurance						
Private	Ref					
Government	3.76	3.17-4.34	<.001	1.32	1.16-1.51	<.001
Other	0.86	2.24-3.89	.109	1.02	0.78-1.33	.874
Low birth weight	28.37	26.30-30.47	<.001	2.72	2.17-3.43	<.001
Prematurity	32.54	30.62-34.49	<.001	1.74	1.40-2.18	<.001
Trisomy 21	7.13	6.02-8.25	<.001	1.74	1.31-2.32	<.001
Turner syndrome	9.80	5.57-14.20	<.001	1.45	0.69-3.06	.319
Trisomy 18	49.75	39.61-60.63	<.001	5.24	2.12-12.97	<.001
DiGeorge syndrome	24.12	21.96-26.33	<.001	1.70	1.23-2.33	.001
Era						
Era 1 (2004-2009)	Ref					
Era 2 (2010-2015)	4.16	3.51-4.82	<.001	0.97	0.8-1.12	.721
Era 3 (2016-2023)	6.93	6.13-7.73	<.001	0.44	0.36-0.54	<.001
Center volume						
Low tertile	4.99	4.22-5.76	<.001	1.14	0.97-1.35	.106
Middle tertile	2.02	1.43-2.62	<.001	0.97	0.85-1.11	.749
Top tertile	Ref					
Red blood cell transfusion	5.40	4.38-6.42	<.001	1.22	1.01-1.47	.037
ARF	29.06	27.10-31.04	<.001	7.06	5.80-8.59	<.001
Pleural effusion	12.56	11.74-13.39	<.001	1.90	1.64-2.20	<.001
Procedures						
Norwood	97.70	94.94-100.50	<.001	18.15	14.06-23.44	<.001
ASO + VSD	59.55	56.78-62.37	<.001	10.44	7.69-14.19	<.001
Truncus arteriosus repair	53.23	50.52-55.99	<.001	6.47	4.69-8.94	<.001
ASO	50.69	48.52-52.90	<.001	4.29	3.03-6.07	<.001
AVSD	10.07	8.96-11.20	<.001	1.47	1.06-2.03	.019
Fontan procedure	34.02	32.72-35.33	<.001	2.67	1.92-3.70	<.001
Tetralogy of Fallot repair	9.18	8.23-10.14	<.001	1.61	1.20-2.16	.001
Glenn procedure	26.31	25.22-27.42	<.001	4.42	3.42-5.72	<.001
VSD repair	Ref					
Off-pump coarctation repair	13.34	12.21-14.48	<.001	1.32	0.94-1.86	.107

Abbreviations: ARF, acute renal failure; ASO, arterial switch operation; AVSD, atrioventricular septal defect; OR, odds ratio; VSD, ventricular septal defect.

### Mortality

There were 1,254/66,695 (2%) in-hospital mortalities, out of those 131/2,672 (5%) occurred in the PCE group compared with 1,123/64,023 (2%) in the no-PCE group (*P* < .001) (Supplemental Table 2). On multivariable analysis, PCE conferred 1.84 (95% CI: 1.50-2.25, *P* < .001) odds for in-hospital mortality (Table 4).

### Follow-Up

Of the 65,441 patients who survived to discharge, 647 (1%) patients had a readmission including PCE within 90 days. Of these readmissions, 541/647 (84%) involved any diagnosis of PCE, 6/647 (1%) had hemopericardium, 27/647 (4%) developed cardiac tamponade, and 73/647 (11%) had a pericardiocentesis. Five-hundred thirty-one (82%) readmissions occurred during the first 30 days after discharge. Readmitted patients due to PCE were older at index operation (8.0 [4.2-36.8] months vs 5.2 [2.2-14.3] months, *P* < .001]) and were less likely to be white non-Hispanic (n = 299/647 (46%) vs 34 223/65,441 (53%), *P* < .001) (Supplemental Table 3).

The procedure with the highest incidence of readmission including PCE within 90 days was the Fontan procedure (n = 138/7,873, 1.7%), followed by the Glenn procedure (n = 113/9,412, 1.2%) and AVSD repair (n = 81/7,633, 1.0%). The procedure with the lowest incidence of readmission due to PCE at 90 days was off-pump coarctation repair (n = 22/6,640, 0.3%) (Supplemental Table 4).

**Figure 3.**
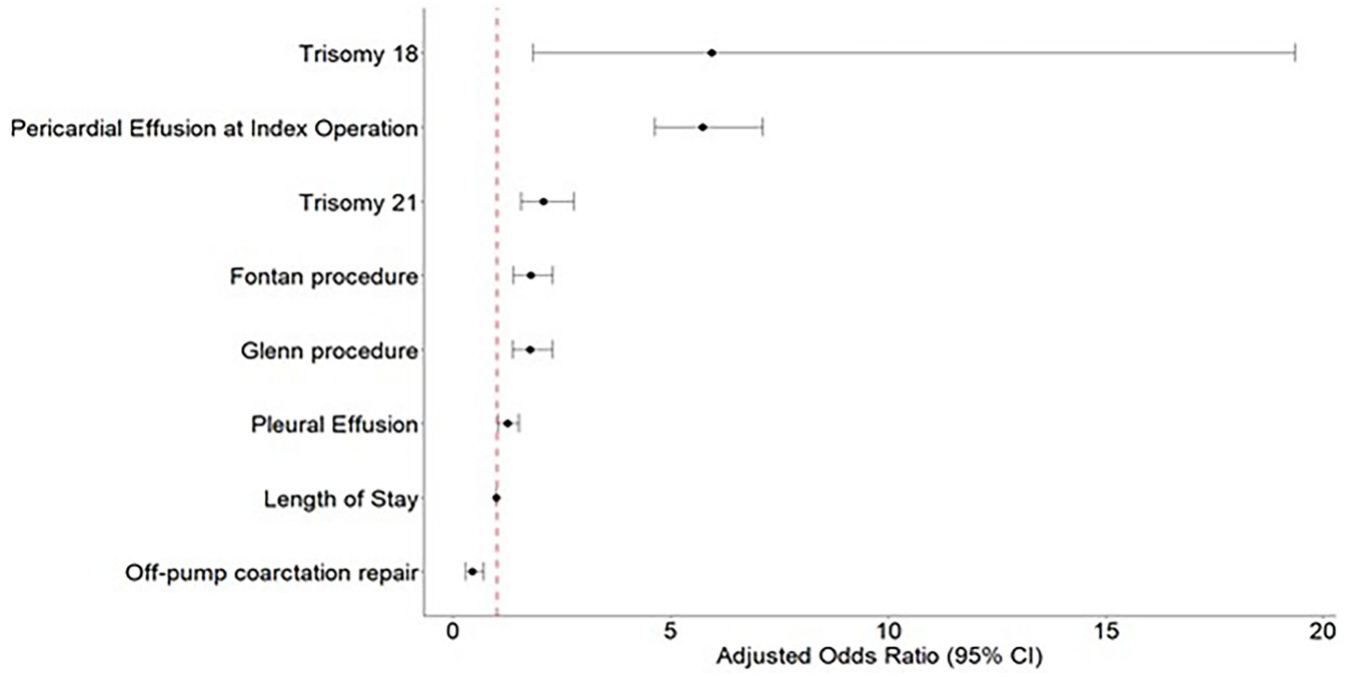
Forest plot: multivariable logistic regression model readmission within 90 days due to pericardial effusion. Reference procedure: ventricular septal defect (VSD) repair.

On multivariable analysis, the Fontan procedure (OR: 1.77; 95% CI: 1.37-2.28, *P* < .001) and Glenn procedure (OR: 1.76; 95% CI: 1.36-2.28, *P* < .001) were associated with increased risk of readmission within 90 days due to PCE when compared with VSD repair. Off-pump coarctation repair was associated with decreased risk for readmission including PCE within 90 days when compared with VSD repair (OR: 0.44; 95% CI: 0.27-0.69, *P* < .001) (Figure 3).

Independent factors associated with increased risk for readmission including PCE within 90 days included, PCE at index operation (OR: 5.73; 95% CI: 4.63-7.10, *P* < .001), older age at indexed operation (OR: 1.00; 95% CI: 1.00-1.01, *P* < .001), and being Hispanic (OR: 1.29; 95% CI: 1.05-1.60, *P* = .015) or Black (1.41; 95% CI: 1.12-1.78, *P* = .002) when compared with white non-Hispanic. Trisomy 21 (OR: 2.07; 95% CI: 1.55-2.77, *P* < .001), trisomy 18 (OR: 5.94; 95% CI: 1.82-19.34, *P* = .003) and patients with a diagnosis of pleural effusion at index operation (OR: 1.24; 95% CI: 1.02-1.51, *P* = .027) also had a higher risk for readmission including PCE within 90 days. Notably, longer LOS at index operation was associated with a decreased risk for 90-day readmission secondary to PCE (OR: 0.99; 95% CI: 0.98-0.99, *P* < .001) (Figure 3).

## Comment

This study utilizes data from the PHIS over 19 years to investigate the incidence and outcomes of PCE following surgical procedures included in the ten STS-benchmark surgical procedures. Among the study cohort, 2,672/66,695 (4%) patients were identified as having PCE during index operation admission. The procedure associated with the highest incidence and risk of PCE was the Norwood procedure. Patients experiencing PCE exhibited a prolonged LOS, increased odds for in-hospital mortality, and increased odds for 90 days readmission secondary to PCE.

In this study, the incidence of PCE was low at 4%, and the procedure with the highest incidence of PCE was the Norwood procedure (n = 245/2,726, 8.9%). The Norwood procedure achieves pulmonary blood flow either through an aortopulmonary shunt (classic Norwood) or a right ventricle-to-pulmonary artery conduit (Norwood-Sano). Although aortopulmonary shunts are associated with a lower PCE risk, heart, and pericardial manipulation during the procedure may still trigger an inflammatory response.^12^ Additionally, the development of seromas around the shunt material has been reported, potentially contributing to PCE.^13,14^ In contrast, the use of a Sano conduit involves more extensive heart and pericardial manipulation, including a ventriculotomy, representing a significant physiological insult.^12^ A higher incidence of PCE may also be associated with prolonged LOS, as patients with extended hospitalizations often undergo more frequent echocardiographic examinations, increasing the likelihood of identifying PCE.

Atrial septal defect repair has been identified in various studies as the procedure with the highest incidence of PCE, whether performed in isolation or in combination with other surgeries.^12,15–17^ While the repair itself may contribute to PCE, it is hypothesized that the underlying mechanism is linked to the degree of left-to-right shunting and the resulting volume overload of the right atrium, which can potentially alter the mechanism for PCE production.^18^ Although isolated ASD repair is not an STS benchmark procedure it is sometimes performed in conjunction with other procedures; as such PCE can be related to this ASD repair. Furthermore, it has been reported that atriotomy and atrial tissue manipulation for intracardiac structure exposure can be the underlying mechanism for PCE development.

As expected, coarctation repair, being an extracardiac procedure and typically performed via thoracotomy, had the lowest incidence of PCE (n = 147/66,695, 2.1%). However, the incidence was not zero, with PCE potentially related to heart and lung mobilization for surgical exposure. In contrast, a previous study has shown that aortic and aortic arch procedures have the highest PCE incidence, though it is likely that those patients underwent additional procedures, with the PCE risk stemming from these associated interventions.^12^ By focusing exclusively on isolated procedures, this analysis provides valuable insights into which surgeries carry the highest intrinsic risk for PCE.

Female gender has been associated with an increased risk of PCE as well as greater morbidity and mortality following congenital cardiac surgery.^2,19^ In our study, female patients exhibited a 10% higher risk of PCE and a 21% higher risk of mortality compared with male patients, aligning with findings from previous research. The underlying causes of the higher incidence of PCE in females remain unclear, although it is hypothesized to be related to differences in immune response.^20^ Further research is needed to explore these mechanisms and identify potential interventions or practice modifications to improve outcomes in this higher-risk patient subgroup.

Patients with Trisomy 21 are particularly noted to have an increased risk for spontaneous PCE, with the underlying etiology unclear but potentially linked to hypothyroidism and increased vascular permeability.^21^ Recent studies have reported that patients with Trisomy 21 and DiGeorge syndrome are at greater risk for PCE after cardiac surgery.^8,12^ However, in this study, neither Trisomy 21 nor DiGeorge syndrome was associated with an increased risk of postoperative PCE. That said, Trisomy 21 patients were found to have a higher risk of 90-day readmission due to PCE. These findings highlight the need for further research to better understand the mechanisms driving the development of PCE in this population and to optimize postoperative care with the aim of reducing the occurrence of this complication.

Pericardial effusion has been associated with increased morbidity and prolonged LOS, although with overall low mortality.^8,22^ This study identified an in-hospital mortality rate of 1.8%, consistent with previous reports.^9^ However, the mortality rate in patients with PCE was more than twice as high, and this association remained significant in a multivariable regression model, where PCE independently increased the risk for in-hospital mortality. The elevated mortality rate among patients with PCE underscores the need for early identification and targeted interventions to mitigate its impact and improve patient outcomes.

In terms of LOS, patients with PCE experienced longer hospital stays, with multivariable analysis showing that PCE was associated with a 16% increase in LOS. These findings underscore that PCE correlates with more complex postoperative care and higher resource utilization, emphasizing the importance of identifying risk factors and implementing effective screening and treatment strategies.

This study found a 1% readmission rate due to PCE within the first 90 days after discharge and this is in line with a previous report where the readmission rate secondary to PCE was reported at 1.1%.^12^ The procedure with the highest incidence of PCE was the Fontan procedure (n = 138/7,873, 1.7%). While no specific etiology has been identified, it is thought to be related to chronic elevations of right atrial pressures, and reports on chronic and recurrent PCE after the Fontan procedure have been published.^23^ These findings emphasize not only the importance of closely monitoring patients during the 90-day postdischarge period but also the need to establish clear follow-up protocols to detect PCE.

## Limitations

The limitations of the current study should be noted. First, its retrospective nature, coupled with the utilization of data extracted from a large administrative database, inherently restricts the ability to establish causative relationships and lacks significant clinical granularity. Second, the lack of a standardized approach to identifying PCE could lead to potential error in estimated incidence, as the definition used is not specific to PCE. Furthermore, PHIS includes only 20% of pediatric hospitalizations and data from just 49 hospitals to date. Consequently, the total number of benchmark procedures analyzed in this study is lower compared to the total number of cases performed nationwide during the same period, which may lead to underreporting this complication.

## Conclusion

In a large multicenter dataset of children undergoing standardized congenital cardiac surgery procedures, the incidence of PCE after CHS is low at 4%. However, certain procedures have an incidence as high as 8.9%. Pericardial effusion is associated with increased mortality and resource utilization. Vigilant monitoring and targeted interventions in high-risk patients are essential for mitigating the impact of PCE on postoperative outcomes.

## Supplemental Material

sj-docx-1-pch-10.1177_21501351251322876 - Supplemental material for Incidence and Outcomes of Pericardial Effusion After Congenital Heart Surgery Supplemental material, sj-docx-1-pch-10.1177_21501351251322876 for Incidence and Outcomes of Pericardial Effusion After Congenital Heart Surgery by Mario O’Connor, Carlos M. Mery, Neil M. Venardos, Jeremy Affolter, Charles Fraser and Andrew Well in World Journal for Pediatric and Congenital Heart Surgery

## Abbreviations

ASD: atrial septal defect
ASO: arterial switch operation
AVSD: atrioventricular septal defect
CHA: Children's Hospital Association
CHS: congenital heart surgery
IQR: interquartile range
LOS: length of stay
OR: odds ratio
PCE: pericardial effusion
PHIS: Pediatric Health Information System
STS: Society of Thoracic Surgeons
TOF: tetralogy of Fallot
VSD: ventricular septal defect
