# Supplementary material for: Incidence and Outcomes of Pericardial Effusion After Congenital Heart Surgery
Source: World J Pediatr Congenit Heart Surg. 2025 Sep 9;17(1):52–60. doi: 10.1177/21501351251322876 (PMC12602724; doi:10.1177/21501351251322876)
Supplement: sj-docx-1-pch-10.1177_21501351251322876 - Supplemental material for Incidence and Outcomes of Pericardial Effusion After Congenital Heart Surgery [file sj-docx-1-pch-10.1177_21501351251322876.docx]

Supplementary Material

Supplementary table 1: Diagnosis and Procedure Codes

| **Diagnosis codes** | |
| --- | --- |
| Pericardial effusion | 4239, I313 |
| Hemopericardium | 4230, I312 |
| Cardiac tamponade | 4233, I314 |
| Acute renal failure | 5845-9, N170, N171, N172, N178, N179 |
| Pleural effusion | 5119, J90, J918 |
| Low birth weight | 764, 7641, 7642, 7649, 76401-8, 76411-8, 76421-8, 76491-8, 765, 76501-8, 76511-8, P0501-8, P0511-8, V2130-5, P0700-3, P0710, P0714-8 |
| Prematurity | 7652, 76521-8, 3622, 36221-7, 76500-26, P0730-9, H35101-3, H35 )109, H35111-3, H35119, H35121-3, H35129, H35131-3, H35139, H35141-3, H35149, H35151-3, H35159, H35161-3, H35169 |
| **Procedure codes** | |
| Blood transfusion | 9900, 9902-4, 30233N0, 30233N1, 30233P0, 30233P1, 30233H0, 30233H1, 30243H0, 30243H1, 30243N0, 30243N1, 30243P0, 30243P1 |
| Mechanical ventilation >96 hours | 9672, 5A1955Z |
| Pericardiocentesis or percutaneous drainage of the pericardium/mediastinum | 370, 0W9C30Z, 0W9C3ZX, 0W9C3ZZ, 0W9C40Z, 0W9C4ZX, W9C4ZZ, 0W9D00Z, 0W9D30Z, 0W9D3ZX, 0W9D3ZZ, 0W9D40Z, 0W9D4ZX, 0W9D4ZZ |

Supplementary table 2: In-Hospital Outcomes

| Variable | Overall  (n = 66,695) | PCE (n = 2,672) (4%) | Non – PCE (n = 64,023) (96%) | p-value |
| --- | --- | --- | --- | --- |
| Length of stay (Days), Median [IQR] | 8 [5.0 – 17.0] | 15 [7.0 – 35.0] | 8 [5.0 – 16.0] | **<0.001** |
| Pre-operative Length of stay (Days), Median [IQR] | 0 [0.0 – 2.0] | 0 [0.0 – 5.0] | 0 [0.0 – 2.0] | **<0.001** |
| Post-operative Length of stay (Days), Median [IQR] | 7 [5.0 – 13.0] | 12 [7.0 – 28.0] | 7 [5.0 – 13.0] | **<0.001** |
| Prolonged Ventilation | 9,718 (15) | 721 (25) | 8,997 (14) | <0.001 |
| Red Blood Cell Transfusion | 5,916 (9) | 319 (12) | 5,597 (9) | <0.001 |
| ARF | 2,168 (3) | 187 (7) | 1981 (3) | <0.001 |
| Pleural Effusion | 10,453 (16) | 718 (27) | 9,735 (15) | <0.001 |
| Mortality | 1,254 (43) | 131 (5) | 1,123 (2) | <0.001 |

Supplementary Table 3. Demographics Patients Readmission During 90 days due to Pericardial Effusion

| Variable | Overall  (n = 65,441) | Readmission 90 days (n = 647) (1) | No Readmission (n = 64,794) (99) | p-value |
| --- | --- | --- | --- | --- |
| Age at Surgery (Months), Median [iQR] | 5.2 [2.2 – 14.5] | 8.0 [4.2 – 36.8] | 5.2 [2.2 – 14.3] | <0.001 |
| Sex |  |  |  |  |
| Female | 28,779 (44) | 301 (47) | 28,478 (44) | 0.203 |
| Race |  |  |  |  |
| White non-Hispanic | 34,522 (53) | 299 (46) | 34,223 (53) | <0.001 |
| Hispanic | 11,494 (18) | 139 (21) | 11,355 (18) |  |
| Black | 8,338 (13) | 109 (17) | 8,229 (13) |  |
| Other | 8,351 (13) | 85 (13) | 8,266 (13) |  |
| Missing | 2,736 (4) | 15 (2) | 2,721 (4) |  |
| Insurance |  |  |  |  |
| Private | 25,623 (39) | 239 (37) | 25,384 (39) | 0.419 |
| Government | 34,933 (53) | 362 (56) | 34,571 (53) |  |
| Other | 4,885 (7) | 46 (7) | 4,839 (7) |  |
| Birth Admission |  |  |  |  |
| Yes | 9,221 (15) | 57 (9) | 9,164 (14) | <0.001 |
| Low Birth Weight |  |  |  |  |
| Yes | 2,410 (4) | 21 (3) | 2,389 (4) | 0.625 |
| Prematurity |  |  |  |  |
| Yes | 3,083 (5) | 23 (4) | 3,060 (5) | <0.001 |
| Trisomy 21 |  |  |  |  |
| Yes | 6,672 (10) | 93 (14) | 6,579 (10) | <0.001 |
| Turner syndrome |  |  |  |  |
| Yes | 284 (0) | 2 (0) | 282 (0) | 1 |
| Trisomy 18 |  |  |  |  |
| Yes | 83 (0) | 3 (0) | 80 (0) | 0.094 |
| DiGeorge syndrome |  |  |  |  |
| Yes | 1,524 (2) | 13 (2) | 1,511 (2) | 0.681 |
| Era |  |  |  |  |
| Era 1 (2004-2009) | 21,936 (34) | 223 (34) | 21,713 (34) | 0.839 |
| Era 2 (2010-2015) | 23,389 (36) | 231 (36) | 23,158 (36) |  |
| Era 3 (2016-2023) | 20,116 (31) | 193 (30) | 19,923 (31) |  |
| Center Volume |  |  |  |  |
| Low tertile | 10,674 (16) | 324 (19) | 10,548 (16) | 0.088 |
| Middle tertile | 20,994 (32) | 126 (30) | 20,797 (32) |  |
| Top tertile | 33,773 (52) | 197 (80) | 33,449 (52) |  |

Supplementary Table 4. Number of Procedures and Incidence of Pericardial Effusion Within 90-days post-discharge

| Procedure | Number PCE | Total Number of Patients | Incidence |
| --- | --- | --- | --- |
| Fontan procedure | 138 | 7,873 | 1.75% |
| Glenn procedure | 113 | 9,412 | 1.20% |
| AVSD | 81 | 7,633 | 1.06% |
| VSD repair | 163 | 16,703 | 0.97% |
| Tetralogy of Fallot Repair | 85 | 9,253 | 0.91% |
| Norwood | 17 | 2,387 | 0.71% |
| ASO | 16 | 2,478 | 0.64% |
| ASO + VSD | 6 | 1,516 | 0.39% |
| Truncus Arteriosus repair | 6 | 1,546 | 0.38% |
| Off-pump coarctation repair | 22 | 6,640 | 0.33% |
